# Foraging with the frontal cortex: A cross-species evaluation of reward-guided behavior

**DOI:** 10.1038/s41386-021-01140-0

**Published:** 2021-08-18

**Authors:** Peter H. Rudebeck, Alicia Izquierdo

**Affiliations:** 1 Icahn School of Medicine at Mount Sinai, New York, NY USA; 2 Department of Psychology, UCLA, Los Angeles, CA USA; 3 The Brain Research Institute, UCLA, Los Angeles, CA USA; 4 Integrative Center for Learning and Memory, UCLA, Los Angeles, CA USA; 5 Integrative Center for Addictions, UCLA, Los Angeles, CA USA

**Keywords:** Reward, Operant learning

## Abstract

Efficient foraging is essential to survival and depends on frontal cortex in mammals. Because of its role in psychiatric disorders, frontal cortex and its contributions to reward procurement have been studied extensively in both rodents and non-human primates. How frontal cortex of these animal models compares is a source of intense debate. Here we argue that translating findings from rodents to non-human primates requires an appreciation of both the niche in which each animal forages as well as the similarities in frontal cortex anatomy and function. Consequently, we highlight similarities and differences in behavior and anatomy, before focusing on points of convergence in how parts of frontal cortex contribute to distinct aspects of foraging in rats and macaques, more specifically. In doing so, our aim is to emphasize where translation of frontal cortex function between species is clearer, where there is divergence, and where future work should focus. We finish by highlighting aspects of foraging for which have received less attention but we believe are critical to uncovering how frontal cortex promotes survival in each species.

## Introduction

All animals, both human and non-human, need to forage in order to maintain nutrient homeostasis. In the wild, animals spend a large portion of their day searching for and then consuming sustenance. While most modern humans do not spend the majority of their day foraging, our human ancestors certainly did. Thus, the need to efficiently find food was a strong evolutionary pressure that selected for specific traits that were then passed down through genes. For each species, their environmental niche would have promoted traits or cognitive abilities that would have increased their foraging success. Thus, appreciating the foraging strategies engaged by a species is essential to understanding what shaped its biology in general and its brain in particular.

Foraging relies upon first assessing where to look for sustenance based on an evaluation of the quality and a prediction of the availability of food. Then behaviors can be planned to maximize food procurement over time. Resource assessment and planning functions are closely associated with functions ascribed to frontal cortex [1, 2]. By understanding how animals typically forage we may be able to glean insight into the functions of frontal cortex across species [3].

The link between foraging and frontal cortex is particularly relevant to current discussions on the relationship between rodent and primate frontal cortex, which is our primary focus in this review. This question is essential for understanding how to interpret the myriad of results from neuroscientific studies of frontal cortex in rats to macaques, which we primarily focus on here. This debate has often focused heavily on neuroanatomy, for instance on the differences in the cytoarchitecture—the layered organization of neurons in an area of brain—of frontal cortex between the species [4]. Here we advocate for a more holistic view that incorporates frontal cortex anatomy within the context of foraging behaviors. In that regard, our approach incorporates neuroethological approaches to cross-species comparisons and is similar to the approach taken by Cisek [5].

Consequently, in this review, we first discuss commonalities and differences in foraging behavior across rats and macaques, but also draw on research from mice and marmosets as well. We believe that foraging niche is germane to understanding the distinctions between frontal cortex in these two animals. Then we delve into the anatomical organization of frontal cortex in rats and macaques. Rather than center our analysis on the evolution of neuroanatomy (which can be found in Preuss and Wise, in this issue) or evolution more generally [5], here we focus on cytoarchitecture and the connections of frontal cortex. In particular, we review recent comparative work that has explored the correspondences and distinctions in frontal cortex connections in rats and macaques [6, 7]. We then turn to comparing functional anatomy, highlighting commonalities in the roles that two parts of frontal cortex, orbitofrontal (OFC) and anterior cingulate cortex (ACC) play in foraging as assessed by reward-guided tasks. We concentrate on these areas as their relationship between rats and macaques is more apparent, although as we will discuss key differences remain.

We finish by highlighting the aspects of foraging that we believe have not received ample consideration and which are markedly different between rodents and primates: foraging range and “time horizon” [8]. Here we do not mean time in terms of the delay to receiving food on the order of seconds but rather in days, weeks, or even seasonal changes in food resources; the kind of information pertinent to foraging success and therefore survival in all animals over the course of their lives. Closely related to the consideration of foraging ranges and time horizons in rats and macaques, we acknowledge there may also be important distinctions and parallels in attention and working memory functions [9]. However, due to space considerations we limit our review to reward-related foraging and results of experiments with that primary assessment in mind.

## Foraging behaviors in rodents and non-human primates

**Fig. 1:**
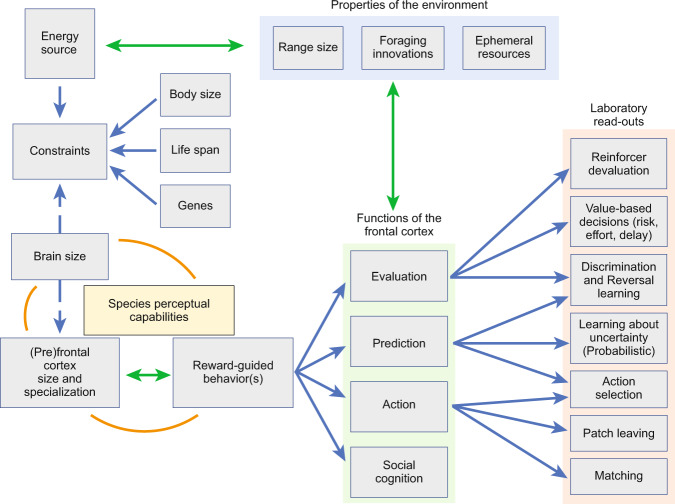
Summary of foraging factors that may contribute to frontal cortex size and specialization. Adapted from [11] to highlight frontal cortex size and specialization as determined by constraints in how the species uses energy (body size, life span, genes), how the species copes with different properties of the environment (foraging range size, ephemeral resources, and emergence of foraging innovations), and modulated by their perceptual capabilities. Functions of frontal cortex in both rodent and primate species include evaluation, prediction, action, and social cognition. The laboratory read-outs of these functions map on to several aspects of frontal cortex and are compared across rodent and primate species in this review. With bidirectional green arrows, we highlight that reward-guided behaviors may have contributed to plasticity in the size and specializations of the (pre)frontal cortex. Also possible are interactions between the properties of the environment in shaping functions (specializations) of frontal cortex, and simultaneously contributing to the energy flow to the organism. An important function of the prefrontal cortex may be to incorporate foraging innovations into the species’ behavioral repertoire. Although social cognition is an important function of the frontal cortex arising from cooperation between groups to forage across long ranges, especially in primates, we do not review the evidence here.

When foraging, all animals, both rodents and primates included, must solve a number of problems to find food. They must perceive and determine food availability, incorporating an assessment of probability, risk, and the cost required to procure the food (i.e., make a Prediction). They must also assess the quality of food among available options (i.e., make an Evaluation). Finally, they must adopt a strategy, or a plan as to how and when to forage, when to explore and seek information about the environment vs. maximizing a known food patch (i.e., plan a set of Actions). These three aspects of foraging have clear associations with concepts linked to stimulus, response, and outcome processes from behavioral learning theory [10]. How different animals solve the prediction, evaluation and action problems, and thus forage, depends on many factors but is heavily influenced by their homeostatic needs as well as their perceptual and physical abilities (Fig. 1).

In primates, there are many explanations for brain evolution ranging from genetic, developmental, social, and ecological to include some aspect of foraging [11, 12]. Recent evidence suggests that meeting the energetic and dietary needs of a rapidly accelerating metabolism [13] was a primary driver in increasing brain-to-body size ratio; perhaps even more influential than the role of increasing social complexity and cooperative group sizes needed for foraging [11]. All explanations for primate brain evolution are based largely on correlational evidence, so there is likely no single mechanism and instead several possibilities for increases in frontal cortex size and specialization, and the innovation of a granular prefrontal cortex, in particular [3]. The need for more efficient and successful foraging played a major role in that development, however what is clear is that rodent and primate lineages diverged 60–70 million years ago, and thus it would be reasonable to assume that differences in frontal cortex enabled adaptation of each animal to their niche.

The most commonly studied non-human primate in neuroscience is the Old World macaque monkey, although New World monkeys such as marmosets are becoming increasingly used. In the wild, macaques’ diet includes seeds, cereals, buds, bark, and fruit. In order to find these foods in ample supply, macaques forage over large distances primarily relying on vision to identify foods. As a consequence, their ranges typically span several thousand hectares [14]. With such a foraging range, perceiving available food stuffs at a distance, planning what type of food to forage for, the time of day in which to do so, and estimating the egocentric distance in accordance with the scarcity and ephemerality of resources are key considerations [15–17]. These abilities are thought to have arisen from the mental mapping requirements of frugivorous species and the greater need for ecological sophistication [15]. Indeed, forming large spatial-temporal maps or mental representations of the foraging range, as well as discriminating and using both visual and olfactory cues to guide selection of fruits at precisely the right times, are crowning features of primate foraging behavior. Other mammals including rodents do “plan ahead” and cache food in anticipation of seasonal changes, and it is believed this ability coevolved primarily with provisioning offspring, preventing theft of high-value foods, and avoiding the “high costs of a variable environment” [18]. Importantly, rats cache over much smaller ranges, time horizons, and throughout a much shorter life span (i.e., fewer seasonal changes) compared to macaques.

A particularly interesting aspect of the foraging environment is the possibility of the emergence of “foraging innovations” [11], when the species samples new foods and/or adopts new strategies to procure foods. These often lead to increases in the energy flow to the organism, and thus success in mastering the unique foraging niche (Fig. 1). Given the above considerations on the need for primates to plan ahead for the right time to harvest (at a much longer time horizon), we surmise that many foraging innovations in the primate arose to enhance the Prediction function of frontal cortex, particularly those contributing to the formation of mental maps used for future planning in space and time. These innovations, resulting in enhanced mental representations, may correspond to specializations of granular OFC, ventrolateral prefrontal cortex (VLPFC), and dorsolateral prefrontal cortex, in particular [3].

Compared to macaques, the foraging ranges and time horizons of rats are much smaller, in accordance with their smaller body size and dependence on odor-guided navigation [19]. Their ranges are also bounded by the assessment of predation risk [20] and the reach of their perceptual capabilities in discriminating the cues associated with that risk. Without prior knowledge of their environment, a rat’s foraging range is the airborne “odor plume” gradient toward the odor source, making stimulus discrimination critical to initial foraging in unknown territories. Yet with increased experience with the locations of the odor sources, rodents switch their foraging strategy to weight knowledge of previous locations more heavily, resulting in a more efficient search strategy [14].

Rat foraging is also affected by indirect, not direct, cues signaling predation risk: the former being more reliable for estimating risk from a wider range of predators than the latter. For example, rodents will collect more food on nights with low moonlight and greater precipitation than in the presence of a specific predator odor [21]. Thus, foraging innovations in rodent species may have arisen in favor of enhancing the assessment of cues in their more immediate environments with short time horizons, or the Evaluation functions of frontal cortex. In summary, though both rodent and non-human primates necessarily engage in Evaluation and Prediction functions for optimal food procurement, mastery of their unique foraging niches likely favored one function over the other. With wide foraging ranges, macaques have to rely on aspects of prediction to increase their chance of finding high-energy foods, whereas in rats, evaluating proximate options likely enhances survival when foraging locally.

The Action function may be most similar across rodent and primate species as it involves a convergence of evidence accumulation pathways to resolve a single outcome among all possible alternatives. Mechanistically, action selection requires interactions of frontal cortex with sensorimotor systems, shaped by adaptation to foraging environments in both rodent and primate species [22]. We review anatomical boundaries of rat frontal cortex and macaque frontal cortex along with their functions, next.

## Comparative brain anatomy

Macaques and rats forage in very different ways, as the prior section highlights. Macaques use highly developed visual perceptual abilities to forage over large ranges to find high-energy foods that are essential to meeting its homeostatic needs; rats rely on acutely developed olfactory abilities to forage more locally. When thinking about how these differences in foraging may relate to neuroanatomy it may be useful to consider an analogy from the world of business.

At their most elemental, large international businesses like Airbus are not so different from your local independent grocery store: both need to balance profit and loss by seeking out paying customers, and both need to conduct this important process of accounting. Despite these similarities, there are meaningful differences in size and economic niche that necessitate different organizational structures. For instance, marketing as well as research and development divisions of a large corporation like Airbus are highly specialized teams, sometimes many hundreds of people who are dedicated to each function. The marketing at your local grocery store might be a single person who also has other duties, whereas research and development may simply not be covered by anyone. Critically, and perhaps most relevant to our topic here, some functions such as accounting can be studied in one company and reliably translated to the other, although such functions may be more distributed in larger companies. To return to comparisons of rat and macaque frontal cortex, given the divergence in foraging niches, differences in frontal cortex organization should be plain to see at the level of cytoarchitecture and connections.

In his parcellation of the human brain in 1909, Brodmann distinguished between the anterior granular and posterior agranular cortex of the frontal lobe [23]. Granular frontal areas are defined by their six-layer structure and clearly visible layer IV made up of small, densely packed granule cells. Brodmann noted that the granular frontal areas, especially on the lateral surface of frontal cortex were present in humans as well as other primate species, but were largely absent in other mammals such as rodents.

**Fig. 2:**
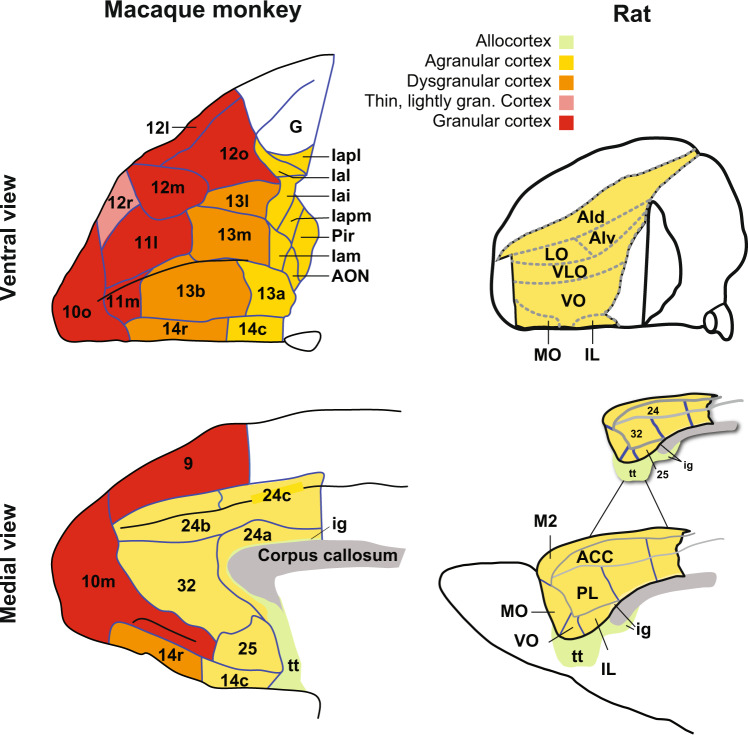
Comparative neuroanatomy of the macaque monkey and rat frontal cortex. Ventral (top) and medial (bottom) views of the macaque monkey (left) and rat (right) frontal cortex. The cytoarchitecture of each area is indicated by different color shading: granular (red), thin lightly granular (pink), dysgranular (orange), agranular (yellow) and allocortex (green). Inset showing areas 24, 32, and 25. Based on connectivity analyses, [33] proposed that the rostral portions of areas 24, 32, and 25 are most similar to primate ACC connectivity. We do not use Cg 1 and Cg 2 nomenclature (a dorsal-ventral distinction) for this reason. Adapted with permission from [125] and [147].

In macaques, later work by Walker and Von Bonin and Bailey showed that caudal parts of orbital, including agranular insula cortex and parts of area 13, and medial frontal cortex, including areas 25, 32, and 24 are agranular, in that they lack the clear granule cell layer [24, 25]. More anterior parts of the macaque medial (10 m), orbital (areas 13, 11, and 12) and lateral prefrontal cortex (46, 9, and 6) are either dysgranular or fully granular frontal cortex (Fig. 2). What the specific function of granule cells are in layer IV and how the presence of granular frontal cortex imbues higher cognitive abilities are unclear, but over time these anterior parts of the primate brain became the defining feature of “prefrontal cortex” [4].

From this initial observation by Brodmann, many articles have been written on frontal cortex organization in primates and other species based on cytoarchitecture [24, 26–30]. Specifically, there has been, and continues to be extensive debate over whether rodents have a region of prefrontal cortex similar to the anterior granular cortex present in primates [31, 32]. Here we accept the view taken by Preuss [4] and later developed by Wise [9] that have addressed this point in depth on the basis of cytoarchitecture.

Frontal cortex in rats as well as mice including prelimbic (PL), infralimbic (IL), anterior cingulate (Cg 1 and Cg 2 subfields, cf. van Heukelum et al. [33]), and all parts of the orbital cortex are agranular (Fig. 2), in that they lack the densely packed granule cells in layer IV [34]. Based on this as well as other factors that we discuss below, Preuss and others have argued that rats do not possess an area anatomically similar to the dorsal and/or lateral prefrontal cortex in macaques. On the other hand, parts of rat caudal OFC and rostral ACC are similar based on cytoarchitecture, due to shared agranular cortex [9]. Thus, there are similarities between PL, IL, Cg 1/2 (canonically these have been designated areas 32, 25, and 24, cf. [33]), and orbital-agranular insula parts of the macaque orbital cortex. Consequently, Wise suggested that researchers should not focus on whether rats have a part of frontal cortex that cytoarchitectonically resembles the anterior portions of macaque frontal lobes as clearly, they do not. Instead, the focus should be on the shared functions that these agranular areas subserve within the context of the unique foraging niche in which the animal finds itself. In other words, it makes sense to use your local grocery as a case study for how a large corporation might run its accounting division (i.e., a ground-level ability that transfers across niches), but not for how it conducts its research and development (i.e., a specialized ability relevant to a niche that requires large foraging ranges).

In addition to cytoarchitecture, the study of connections of frontal cortex to other parts of the brain is another approach for comparing between rodents and non-human primates (see Haber et al. in this issue, [6, 7, 33]). One of the defining features of prefrontal cortex in macaques and other primates is its bidirectional connections to the mediodorsal thalamus, MD, [35]. Rat frontal cortex is similarly connected with this part of the thalamus [34]. Indeed, on the basis of these connections to the MD, Woolsey et al. proposed that rats have a prefrontal area similar to those in macaques [36, 37]. However, with the advent of better anatomical tracing techniques, researchers have come to appreciate more diversity in these projections [38], and that many other areas outside of prefrontal cortex, such as supplementary motor area and premotor cortex also receive input from MD [39–43], limiting the usefulness of this criterion [4].

Comparison of connections and their physical arrangement is still a powerful approach for understanding differences and similarities of frontal cortex across species. Each subregion of ACC sends projections to distinct parts of the striatum [44, 45]. In a recent series of studies, Heilbronner et al. compared the organization and topography of these projections across rats and macaques from a large database of tract-tracing studies [46]. Their analysis showed that there was concordance between the connections from area 32 and area 25 and to a lesser degree orbital areas to striatum across species, complementing previous analyses [9]. Notably, the connections of area 24 to the striatum in macaques were different to those from the analogous area in rats [46], which is somewhat unexpected since ACC in both rats and macaques is agranular (i.e., the connections would be expected to be comparable if cytoarchitecture is similar). Despite this difference, van Heukelum et al. [33] recently emphasized that based on connections with amygdala, OFC, hippocampus, hypothalamus, thalamus, and retrosplenial cortex, rat, macaque, and human cingulate cortex appear to have a similar rostral-caudal pattern of connections whereby ACC is distinct from mid cingulate cortex. Thus, while cytoarchitecture may not bear as much similarity between species, there is often closer correspondence revealed by connectivity analyses.

Given the prior discussion emphasizing that there are potential anatomical similarities between caudal orbital and parts of medial frontal cortex in non-human primates and rodents, a word of caution is warranted. Even when there are strong anatomical similarities between species, there may still be differences in how a part of frontal cortex contributes to behavior. A recent set of findings from marmosets illustrates this point [47].

One of the classic findings in behavioral neuroscience relates to the roles of rat frontal cortex areas PL and IL in defensive threat conditioning [48–50]. Based on the pioneering work of Quirk and others we known that PL is essential for generating appropriate defensive responses to threats, commonly measured using tone-foot shock pairings. By contrast, IL plays a specific role in storing memories related to threats especially during extinction [51]. When Wallis et al. conducted the equivalent set of experiments in marmosets using loud noise instead of foot shocks, however, they found the opposite pattern of effects to those in rodents. Specifically, inactivation of area 32 was associated with altered memory of extinguished threat associations, whereas inactivation of area 25 impacted expression of defensive responses. This difference between the functions of area 25 may relate to slight differences in connections to structures with direct control over autonomic effectors [52]. Thus, even though there is strong anatomical data indicating that PL and IL in rats are analogous to areas 32 and 25 in non-human primates respectively, it does not always follow that these parts of the brain contributed to behavior in the same way.

Confirming functional similarities is therefore essential for frontal areas that we think may be anatomically similar. We take up this point below with an in-depth comparison of orbital and anterior cingulate areas in rats and macaques concentrating on the key processes engaged during foraging: Prediction, Evaluation, and Action. In making these comparisons we directly contrast orbital and cingulate areas that we have emphasized are agranular in rats and granular, dysgranular and agranular in macaques. This is not because we do not think that anatomical differences are important, but because: (1) while frontal cortex expanded and became more differentiated in macaques compared to rats, the core functions that areas subserved are largely maintained; and (2) though multi-species assessments of foraging behavior do exist [53], direct comparisons of the importance of areas in frontal cortex of rats and macaques are few and far between. For instance, there is only one published study reporting the effects of agranular insula lesions in macaques [54]. Consequently, in this review we attempt to piece together patterns of functional relevance across rats and macaques, using the available literature as well as findings from mice and marmosets.

## Dissecting reward-guided behavior and decision making: Prediction

As we emphasized earlier, animals must predict the availability of rewards when foraging. In this section we focus on this process to reward procurement. In particular, we highlight cross-species comparisons across tasks probing the roles of OFC and ACC, major subregions of frontal cortex. Though some paradigms incorporate aspects of the Evaluation and Action components of foraging, the tasks described here mainly involve discrimination of reward-predictive stimuli, adapting to changes in, and assessment of the uncertainty or risk of not receiving reward.

### Stimulus-based reversal learning

Adaptive foraging relies upon the ability to discriminate stimuli that predict food from those that do not, and to flexibly update behavior when those predictions are violated. In other words, an animal needs to keep track of food availability. This ability has been classically measured in laboratories using discrimination and reversal learning paradigms. There are many variants of this paradigm that have been comprehensively reviewed including stimulus, action, and spatial versions and with stimuli presented in different sensory modalities [55–57]. Here we focus our discussion on stimulus-based versions of the task as they may pertain more closely to learning about reward-predictive cues in foraging niches and have been extensively studied across rodents and primates.

In one commonly-administered paradigm, subjects are presented with stimuli or three-dimensional objects, one of which results in reward every time it is chosen (e.g., selecting S_A_ results in reward compared to S_B_ or S_C_…), wherein the subject learns about the sensory features of the stimulus that bring about reward and those that do not [58–63]. After reaching a performance criterion, a set number of trials/errors, or consecutive wins, the stimulus-reward contingencies are reversed. It is important to note that while mice, rats, and macaques are able to complete discrimination reversal tasks, performance varies widely. While macaques often take a few trials to change their responding the first time they experience a reversal, rats and mice can take many hundreds of trials [55]. This difference illustrates an instance where non-human primates differ from rodents in their abilities to predict changes in rewards.

The neural systems involved in reversal learning have been reviewed in detail elsewhere [55], so we focus instead on two regions of frontal cortex, ACC and OFC, and direct comparison across rats and macaques. The role of OFC in behavior is inextricably linked to reversal learning. In nearly every species tested, damage to this area is associated with decrements in performance on reversal learning tasks [64]. Recently, however, with new findings in macaques, the contribution of OFC to reversal learning has become unclear. This is because in rhesus macaques, unlike large, aspiration lesions of OFC [65], excitotoxic fiber-sparing lesions of OFC including Walker’s areas 11,13, and 14 do not adversely impact fully-predictive (deterministic) reversal learning [66, 67]. Smaller excitotoxic lesions of subregions of OFC or anterior agranular insula cortex similarly do not impact performance of reversal learning [54, 66]. Importantly, when a narrow “strip” lesion of posterior OFC was made (incorporating posterior parts of areas 13l, 13m, 13a, and 14c, see Fig. 2) this reproduced the reversal learning impairment seen after aspiration lesions of whole OFC. This indicates that these “strip” lesions interfered with white matter projections, such as the uncinate fascicle that course into the ventral frontal lobe around this point.

In contrast, excitotoxic lesions of OFC areas 11 and 13 in marmosets *do* produce reversal-specific effects [68, 69]. This effect is similar to the impairments on analogous stimulus-based reversal learning with deterministic rules that have been reported in rodents with excitotoxic lesions of OFC [61, 63]. While it is difficult to provide a simple account for these patterns of effects in two species of non-human primates as well as rats after OFC lesions, we surmise that this divergence could stem from two related features. First, compared to New World monkeys such as marmosets, Old World monkeys have an enlarged and more highly differentiated prefrontal cortex [70]. Second, while marmosets have advanced visual abilities compared to rodents, their foraging ranges are much smaller than macaques and their primary food sources, including tree sap and insects, necessitate more local foraging [71, 72]. A third possibility that could be tested experimentally is that marmosets and rodents use similar strategies to learn the discrimination reversal learning task. By contrast, macaques either use a different strategy or can rely on multiple different aspects of prediction to quickly reverse the associations between stimuli and rewards. The combination of these factors thus influence the involvement of OFC in reversal learning, and may be related to foraging strategies.

By contrast to OFC lesions, damage to ACC (areas 24) primarily impacts both action-based reversal learning in macaques, but deficits in stimulus-based tasks are also apparent [73, 74]. Impairments revolve around maintaining appropriate Win–Stay/Lose–Shift-like strategies across trials, which we review later. Lesions of ACC and posterior cingulate in rat are without effect on fully-predictive reversal learning [75]. Interestingly, both perigenual ACC (area 32) and OFC (area 11 and 13) in marmoset are necessary for related visual stimulus-based reward contingency learning (i.e., tested by the extent to which one pair of actions and its outcome can be selectively degraded) [76], suggesting that there may be shared support of this function in primate frontal cortex. Though there are no equivalent data in rodent that we know of, we hypothesize that there would be a similar shared division of labor of these regions in rodents. It would be interesting to assess if the pattern diverges in Old World macaques. Indeed, species differences pull apart when more prediction is required (i.e., in probabilistic paradigms), which we discuss in more detail below.

### Probability and risk-based decisions

It has long been known that patients with OFC lesions perform poorly on gambling tasks where the association between choices and reward is probabilistic [77]. Neuroimaging studies have consistently found that uncertainty about reward is correlated with BOLD signal in human OFC (for instance, Hsu et al. [78]). Thus, there is ample evidence that human OFC also encodes risk of reward. With intracranial recordings Li et al. [79] found that both medial and lateral OFC signal reward probability in humans. Importantly, although human ACC has been implicated in linking reward information with alternative actions/options [80], and risk prediction, or “expected risk” [81], it has not been demonstrated to have causal involvement in risk or probabilistic decision-making per se.

Macaque experiments are largely consistent with these observations: neural populations in monkey OFC signal the variance of different probability distributions of reward [82] and a similar “expected uncertainty” signal in ACC (area 24) has also been reported [83, 84] although there appears to be much more diversity and valence-specificity in ACC (e.g., reward, punishment, and uncertainty), rather than a general risk signal, as in OFC. The function of this variance/risk encoding or expected uncertainty is not completely known though there are several theoretical perspectives on the topic [85–87], but this could enable adaptation to changes in the value range of options (i.e., range adaptation), which has already been studied in the context of economic decisions, and due to space limitations we do not review here [88, 89]. Range adaptation has been shown to involve primate OFC [88], therefore, there is an abundance of data to suggest that there are risk representations in both ACC and OFC, in both human and non-human primates.

Unfortunately, few studies have probed this kind of known risk or expected uncertainty in rodents with few exceptions, c.f. “fixed relative uncertainty,” or SD, [90, 91]. Instead, the most popular behavioral paradigm in rodents involves less prediction (i.e., learning about individual option values) and more evaluation (i.e., choosing between known values). Typically, these tasks involve animals choosing between options associated with different reward probabilities wherein the likelihood of reward is varied for one option. For this type of task, rats are presented with a lower probability large reward vs. a higher probability small reward, deemed risky vs. safe options, respectively. Similar to effort- and delay- discounting paradigms, the preferred, larger reward is associated with at-first equal probability of 100%, but then decreasing probabilities of reward through subsequent blocks of trials. Such probabilistic discounting assays have been utilized to explore dissociations within rodent frontal cortex in risk, concluding that PL, but not OFC, is involved in probabilistic discounting [92, 93], with dopamine in OFC similarly not involved in this kind of decision making in rats [94].

Recent studies have uncovered that transient inactivations of medial, and not lateral OFC, render rats more risk-preferring [95]. However, there are other tasks, as noted above, that do not employ discounting methods where probabilities vary by block, but instead require animals to first learn the probabilities and offer no “safe,” predictable rewards. Under these conditions rats with lesions to (predominantly lateral) OFC exhibit a clear preference for risk [96], consistent with the results for macaque OFC. Rat OFC is key for generating value expectations [97], and perhaps value distributions or risk [91], that may be used to determine whether value updating is needed or not [98]. Though there is significant evidence that ACC plays a role in evaluating predatory risk [99], there does not appear to be strong evidence of a prominent role for ACC in reward risk in rats, as measured by probabilistic discounting [93]. To our knowledge, encoding of such variance or expected uncertainty has not been reported in rats. In sum, assessing whether rat and macaque studies converge in probabilistic and risk-related decision making is unclear and requires further investigation. One key aspect of such efforts would be to better match behavioral paradigms across species.

### Stimulus-based reward learning under uncertainty

Deterministic reversal learning tasks have been useful for gaining initial insight into how parts of frontal cortex make predictions about reward availability but in the wild, rewards are rarely available with perfect certainty. Instead, most animals have to incorporate the probability of obtaining food into their predictions. Further, this assessment of food availability has to be constantly updated as food is procured (or not). Citing evidence from both rat and macaques, we recently reviewed how stimulus-based variants of probabilistic reversal learning (PRL) can serve as a powerful animal model for investigating neural mechanisms of reward learning under uncertainty [86]. For example, neural recording experiments have revealed that activity in OFC and ACC is correlated with behavior during probabilistic and reversal learning [100–105]. Studies using pharmacological inactivation, lesion, and virally-mediated approaches have further confirmed these roles in probabilistic reward learning [106–109] and have helped to dissociate the functions of each structure in this type of learning.

Consistent with the evidence reviewed above, OFC recordings in monkeys [110] suggest largely orthogonal representations—or single-variable encoding—of reward attributes (i.e., probability, magnitude, volatility). Importantly, the timing of encoding for each of these attributes is simultaneous, suggesting there is parallel encoding of these attributes in OFC [110]. Furthermore, representations of subjective value in OFC are transient and not integrated across the length of time that would be required for decisions [111]. Instead, such temporal integration may instead occur in ACC neurons [112], where perhaps global learning rates are also set [87]. Though not specific to ACC, neurons in the medial frontal cortex of mouse similarly bias choices with slow decay [113], suggesting there are stable representations of value in this region as well. Supporting this, an analysis of population and single-unit activity across various regions of macaque PFC [112] suggests that ACC is important in accept/reject decisions, and while ACC contains some information that is redundant with that encoded by OFC (e.g., stimulus value), it also contains additional information not encoded by OFC, such as action values. This specialized role for ACC in action selection and strategy is a point we take up below in “Dissecting reward-guided behavior and decision making: Action” section.

Despite the discussion above, precisely which parts of the ventral prefrontal cortex in non-human primates are essential for learning and tracking probabilistic reward associations has recently become more clear. Previously, OFC appeared to be central for this aspect of behavior: aspiration lesions in non-human primates and excitotoxic lesions in rats both impaired probabilistic reward learning (for example, [114, 115]). However, subsequent work showed that excitotoxic lesions of OFC in macaques, including Walker’s areas 11, 13, and 14, are without effect on PRL, but do impact reward assessment in reinforcer devaluation settings [116]. Instead, lesions of the more laterally adjacent VLPFC, including areas 12, 45 and ventral 46, impair probabilistic reward learning, but not reinforcer devaluation tasks. This specialized role for VLPFC in probabilistic reward learning is consistent with findings from fMRI [117], aspiration lesions of VLPFC [118], and pharmacological studies in macaques [119]. It also corroborates emerging work in humans [120].

While the difference between the contribution of lateral OFC and adjacent VLPFC to probabilistic learning might seem trivial because both are on the orbital surface of the frontal lobe in macaques, such a view misses a number of key points. First, it is important to realize that studies of macaques with OFC lesions have almost never included VLPFC, Walker’s area 12 (for example, [65, 114, 121]. Instead, VLPFC was seen as separate to OFC, as area 12 which spans both orbital and lateral surfaces is distinct in its cytoarchitecture from areas 11 and 13 [24]. Second, aspiration lesions of VLPFC produce deficits on deterministic reversal learning tasks that are distinct to those following OFC lesions [122]. Taken together, VLPFC appears to be specialized for probabilistic learning and its function appears to be distinct from OFC [123].

If VLPFC, not OFC, in macaques is critical for probabilistic reward learning, then how does this relate to rat frontal cortex? The part of the rat frontal cortex closest in location to VLPFC is lateral OFC, and as we noted above, this area and circuit incorporating it are essential for (spatial-based) probabilistic learning [108, 115]. As we review in “Dissecting reward-guided behavior and decision making: Evaluation”, lateral OFC in rodents is also required for evaluative processes during foraging as measured by devaluation tasks [124]. As such, lateral OFC in rats appears to support both of the functions separately ascribed to OFC and VLPFC in macaques. One account of these findings is that the more lateral parts of rat lateral OFC, such as the dorsal-lateral area (DLO), might be specialized for probabilistic learning [108]. Although we note that more posterior lateral OFC lesions in rats do impact deterministic reversal learning, it is unclear how this might translate to probabilistic reward settings.

It is also important to realize that whereas there are clear anatomical similarities between rat and macaque OFC, none exist for VLPFC as this granular frontal area emerged during non-human primate evolution (Fig. 2, [28]). Unlike OFC, which receives inputs from multiple sensory modalities and is preferentially connected to rhinal cortex, VLPFC primarily receives input from inferotemporal cortex [125–127]. This distinction means that unlike the rich and varied sensory information arriving at OFC, VLPFC primarily receives visual information below the level of whole objects. As Murray et al. highlighted, VLPFC emerged in anthropoid primates as it likely provided a selective advantage for estimating the availability of food at distant locations, a mode of foraging that became especially important as non-human primates became larger and their foraging ranges became more extended [128]. When looking for food at distant locations, information about the fine-grained visual properties, smell, or taste are less useful. As we have speculated before [116], a role for VLPFC in representing reward probability may have arisen because this area provided an advantage (a “foraging innovation”) in estimating resource availability at distant locations. This point highlights that compared to rats, the different foraging niche of macaques required more highly developed prediction of food, especially those at distant locations.

## Dissecting reward-guided behavior and decision making: Evaluation

Unlike making a prediction about where or how to forage, the Evaluation aspect of foraging is more dependent on a local assessment of a potential food. For instance, from outside of the grocery store you might be able to see that they have strawberries, but it is only when you go inside and get close enough to closely inspect and smell them that you can evaluate whether they are good to eat. In laboratory settings, the evaluation of options has been assessed with a number of different paradigms including reinforcer devaluation, specific Pavlovian-to-instrumental transfer, incentive learning, and over-expectation tasks to mention just a few. Of these, reinforcer devaluation has been most extensively studied across rodents and non-human primates. These tasks not only test whether a subject can remember the sensory qualities of a reward, such as its smell and taste, but also whether they are able to update and use the current value of the food to guide behavior. This ability to update the desirability of a food is often done by manipulating its value by associating the food with sickness or satiety [129]. Notably, in both rats and macaques performance of reinforcer devaluation tasks is qualitatively similar: both rats and macaques are able to rapidly update the value of an association after satiety or sickness and use that knowledge to guide choices [129, 130]. In this regard the evaluative aspect of foraging appears to be similar between rats and macaques.

Over many years and experiments a general consensus has emerged that OFC in both rats and macaques is essential for appropriately evaluating options in the reinforcer devaluation task (for instance, Izquierdo et al. [65], Gallagher et al. [124]). Specifically, without a properly functioning OFC, monkeys and rats are unable to appropriately change their behavior to track the current value of a particular food. Similarly, recordings in OFC in devaluation settings show that neurons in this area track the current value of rewards [131, 132]. Similarly, work in humans shows that this role for OFC in evaluating the quality of foods is well-conserved across species [133, 134]. Furthermore, in all of the species tested, the evaluation of potential foods requires functional interaction between OFC and amygdala [135–137]. This role for OFC in evaluation of the sensory specific value of food items in rats and macaques fits well with its dense connections from sensory and limbic structures [138]. Indeed, recent work has highlighted the association between sensory cues as being the key mechanism engaged in OFC [139].

There are, however, a number of differences in functional anatomy between rats and macaques with regard to the evaluation of foods during devaluation tasks. In rats, initial insights into the parts of frontal cortex engaged in devaluation tasks highlighted a role for PL in the medial frontal cortex [140] as well as OFC [124]. One interpretation was that PL and OFC were essential for devaluation in instrumental and Pavlovian settings, respectively, with lateral OFC especially necessary for updating Pavlovian associations [141]. This difference between Pavlovian and instrumental based settings is consistent with the effects of OFC and ACC lesions in macaques in stimulus- and action-based tasks [142].

When Rhodes et al. directly tested the role of macaque area 32, the area analogous to area 32/PL in rat frontal cortex [33], in an instrumental devaluation task they found that lesions were without effect [143]. Previously, area 32 lesions had also been shown to be without effect on stimulus-based devaluation tasks, indicating that area 32 is not involved in updating and retrieving the current value of foods [73]. By contrast, in the instrumental devaluation tasks, lesions of macaque OFC did impact ability to update their evaluation of potential rewards after sensory specific devaluation. Follow up studies again confirmed the specificity of the macaque OFC-amygdala pathway for reinforcer devaluation, but extended these to instrumental settings [144]. Thus, in macaques, OFC is essential for the evaluation of potential foods, whereas in rats these functions appear to involve both OFC and PL [140]. It remains to be determined, however, if more dorsal area 24 in rat serves a similar function. Such a difference between rat and macaque may reflect differences in OFC connections in primates [138], or it may reflect a priority for Evaluation functions to be better integrated into assessments of foraging in rodents, where local evaluation of food items dominates behavior.

OFC is not, however, a homogeneous structure [145] and in particular there are major differences in connections over the medial-lateral and anterior-posterior extent in both rats and macaques [125, 146, 147]. Across the medio-lateral extent, key differences in connectivity to subcortical structures are apparent [148, 149]. Extant research indicates that lateral OFC in rat and macaques is specialized for choices between objects or stimuli [66, 150], whereas medial OFC, especially anterior medial OFC in rats may, like PL, play a role in updating and using reward value in instrumental settings [151, 152].

As we noted earlier, interaction between amygdala and OFC is central to performance on devaluation tasks and amygdala projections to OFC exhibit a gradient from posterior to anterior, with the highest density of amygdala inputs in posterior OFC [153]. This indicates that there may be differences in how anterior and posterior lateral OFC contribute to evaluating different foods in devaluation tasks. Recently, Panayi and Killcross [154] found that lesions of either anterior or posterior lateral OFC impacted the performance of rats in Pavlovian devaluation tasks. Adaptive performance in these types of tasks, however, requires two distinct processes; updating the value of currently available foods and then using that stored value to guide responding. Based on lesion experiments OFC is required for both, whereas amygdala is only required for the updating of values [155], which is in contrast to pathway-specific manipulations showing that amygdala terminals in OFC, but not OFC terminals in amygdala, are crucial for adaptive conditional responding [135]. The experiments conducted by Panayi and Killcross [154] were not designed to distinguish between updating and retrieval processes and the different contributions of anterior and posterior OFC. However, in a recent study in macaques, Murray et al. were specifically able to address this question using temporally specific inactivation of anterior and posterior OFC in macaques. They showed that pharmacological inactivation of anterior lateral OFC, primarily encompassing Walker’s area 11, was important for using the stored value of a particular food reward after it had been updated. Posterior lateral OFC was found to be essential for updating the value of a food, but not subsequently using that updated value. Thus, distinct parts of non-human primate OFC appear to be important for updating and using the stored value of a reward to guide behavior.

While a similar dissociation between anterior and posterior OFC in rodents during devaluation paradigms has not been reported, a recent study by Malvaez et al. did find a dissociation between updating and storing the current value of a food reward in OFC during incentive learning. This study assessed how projections from lateral or medial OFC to amygdala contributed to updating and retrieving the value of reward to guide rats’ behaviors [156]. Using a combination of carefully designed behavioral tasks, glutamate recordings, and chemogenetic manipulation, they showed that projections from lateral OFC to basolateral amygdala are essential for updating reward value. Interaction between medial OFC and amygdala was essential for using the stored value of a reward to guide current behavior. Note that this is potentially different to macaques where anterior lateral OFC is more important for the using the current value of reward to guide behavior. Drawing such a distinction is premature, as the incentive learning task used by Malvaez et al. entailed instrumental responses, and it is unknown how medial OFC in macaques might contribute in these settings. Ultimately, further experiments are required to determine how OFC in rats and macaques contributes to the evaluation of different food rewards. What is clear, however, is that both agranular OFC in rats and granular OFC in macaques are central to the evaluation of foods during foraging that can only occur when the associated sensory features of a food can be inspected closely and a decision to eat or not is made.

## Dissecting reward-guided behavior and decision making: Action

At the same time as predicting and evaluating different potential foods, animals have to determine the set of actions or strategy that will enable them to obtain those foods. ACC is a key integrator of reward, cognition, and action planning [1, 2, 157, 158]. Notably, neurons in this area track trial-by-trial outcomes of choices [159–164], reward history [165], reward prediction errors, RPEs [100–102, 162], and represents better, unobtained rewards [166]. These counterfactual outcomes or “fictive reward signals” [161] may be especially informative in integrating multiple decision parameters allowing the comparison of alternative responses in ACC so that more fruitful actions can be taken. A role for the ACC in volitional control action is also underscored by its connections to parts of the brain involved in preparing and executing actions including premotor cortex as well as motor effectors in the brain stem [167].

ACC may not contribute robustly to probabilistic, stimulus-based reversal learning in rats [168] or macaques [73]. Instead, many of the “low-level” stimulus-based OFC-encoded variables described above may be multiplexed in ACC [166] to perform computations that derive higher-level signals such as RPE [101], confidence (or conversely) uncertainty of predictions [168], with mixed neural selectivity of these variables yielding increased dimensionality [169, 170]. These derived signals could provide a way to monitor overall performance and update behavioral strategies when necessary (particularly overall trial strategy following positive feedback, i.e., Win–Stay [168, 171]).

Several groups have shown that ACC neurons in macaques represent unsigned RPE during tasks that mimic foraging-like behaviors, and the RPE signal is correlated with behavioral adjustment [100, 172, 173]. Note that these RPE signals can only occur after a food has been obtained which again indicates that representations generated in ACC occur in a later stage of processing than both prediction and evaluation functions highlighted earlier. Similar to macaques, recording neural activity in rat ACC, Hyman et al. [101] found neurons in this area encode past outcomes and, when expectations are violated signal negative RPEs, signals that are likely critical for driving behavioral adjustment. It important to note that RPE encoding in ACC are distinct; there is scant evidence that OFC signals RPEs [174–176]. This correspondence between rats and macaques indicates that ACC may play similar roles in both animals, and is essential for updating action plans after food has been procured (or not).

Lesion, pharmacological inactivation, or chemogenetic inhibition studies of ACC point to a causal role suggested by the single neuron recording studies [73, 74, 83, 163, 168, 171, 177], that ACC is required for the maintenance of an action strategy. For example, Tervo et al. [171] found that muscimol inactivation or chemogenetic inhibition of ACC increases behavioral variation, and makes animals more exploratory and less sensitive to feedback. Corroborating this, we have found that DREADDs inhibition of ACC similarly decreases a Win-Stay strategy [168]. This is similar to reports in monkeys, where aspiration lesions of ACC (area 24) in macaques result in impaired use of rewarded trial feedback to sustain the selection of the correct response during learning [73, 74] and pharmacological inactivation of ACC (area 24) in marmosets impacts performance in contingency degradation tasks [177]. This pattern of effects was interpreted by Chudasama et al. as indicating that ACC is essential for “using information about reward and non-reward to sustain effective choice behavior.”

Lesions and transient/reversible pharmacological inactivation of OFC in rats and macaques result in more specific deficits in learning and performance under conditions of probabilistic (risk) and reinforcement uncertainty [91, 115, 116, 145, 151, 178–180], and seem centered not on trial-by-trial strategy but in overall acquisition of the reversal state of PRL, and specific to early phases of learning. In contrast to ACC, lesions of OFC are without effect on action-based reversal learning tasks in macaques [142], indicating that OFC is especially involved in stimulus-based assessments of value, computations that are essential for both prediction and evaluative aspects of foraging. As a notable difference, most PRL paradigms in rodents employ action- and spatial-based reversal learning procedures (with exceptions to the rule, for instance [181, 182]); and OFC inhibition or lesions do in fact impair performance on action-outcome reversals [108, 115, 183]. Thus, support of action- and stimulus-based PRL is potentially a general function of OFC in rats, not specialized as in macaques.

Taken together, this distinction between OFC and ACC indicates that they have dissociable, yet complementary roles in behavior in rats and macaques. Specifically, OFC supports the updating and use of stimulus values. In rats, the estimation of probabilities cumulatively over multiple trials to provide a baseline for computing a need for update/behavioral change within a state is also supported by OFC, but in macaques this function is dependent on VLPFC/12o. By contrast, ACC may instead carry a spectrum of signals encoding learning rates or transition rates [87, 184, 185]. Such representations are essential for estimating reward expectations but also allows violations of these expectations to be signaled promoting changes in actions when violations are detected [86]. In sum, given the proximity of ACC to motor output, both stimulus and action values may be multiplexed at the level of ACC in both rats and macaques to drive choice strategy. One key difference between rats and macaques is also apparent; while prediction, evaluation, and action aspects of foraging rely on single subregions or circuits within frontal cortex in macaques, these processes in rats are subserved by a more integrated set of frontal cortex areas. For instance, OFC neurons in macaques rarely encode spatial locations [186], whereas in rats and mice they encode the spatial location itself [183, 187].

## Conclusions and future directions

Efficient foraging is essential to survival and relies on frontal cortex. Rats and macaques, experimental animals commonly used in neuroscience, occupy unique ecological niches that necessitates different types of foraging. Here we emphasize that appreciating the foraging niche of an animal is essential for understanding the organization and function of their frontal cortex. Variations on this idea have a long history in both biology (for instance, [188]) and neuroscience [5, 128]. Here we emphasized that this point has been overlooked when attempting to draw comparisons or translate findings from frontal cortex between species [31]. As we have seen there are key differences, but also some similarities in how frontal cortex of rats and macaques is organized and contributes to behaviors necessary for foraging. Much is still to be discovered, but appreciating the points of intersection and divergence will facilitate the type of translation essential for furthering neuroscience research as well as enabling medical advances.

When discussing the role of different parts of frontal cortex in forgaing, we primarily focused on a specific set of laboratory based paradigms. This has enabled insight into certain aspects of foraging but has left others understudied. As examples, as we outlined in the “Foraging behaviors” section, planning for the best time of day to forage for foods while estimating spatial distance and accounting for ephemerality of those resources requires higher-level planning [15]. These functions became more advanced in non-human primates, such as macaques. In particular, areas of primate frontal cortex (VLPFC, area 12o, specifically) appear to be more specialized in predicting the availability of foods at distance [3], the probability of reward [116], and the motivation to resolve uncertainty of both rewards and punishments [189]. When foraging at distance macaques have to be able to use both visual information as well as memories of unseen options to guide foraging. Spatial maps or mental representations of the animal’s range likely increased foraging success by enhancing the Prediction of food availability in primates. Consequently, establishing how parts of the brain involved in spatial navigation [190] interact with parts of frontal cortex during foraging would enable this to be revealed. Indeed, the recent characterization of value fields in macaques hippocampus opens the possibility that representations exist [191]. In this regard, the tasks designs for macaques as well as human experiments stand to gain from the many years of task development in rats and mice to probe how spatial cognition and value interact.

We also proposed that smaller ranges and shorter time horizons in rodent reward environments may have produced foraging innovations related to the close-inspection of stimuli or cues involved in the Evaluation of foods. While there is mounting evidence that OFC is specialized for this function through connections with amygdala, other parts of rat medial frontal cortex are also essential, highlighting that evaluation of local items appears to be more integrated across areas in rat frontal cortex compared to macaques. Within rat frontal cortex, lateral OFC appears to be specialized for choices between objects/stimuli, whereas medial OFC may play a larger role in updating and using value and instrumental choices. Another area for future study we highlighted above is in stimulus-based risk and probability learning in rats and macaques. Specifically, better aligning paradigms so as to better ensure tasks do not rely on probability discounting or action/spatial response methods [108], will enhance the translation of findings.

In summary, we outlined important divergences in foraging niches between rodent and primate, which in turn would produce different kinds of problems that frontal cortex is required to solve. Specifically, we highlighted that these foraging differences likely affected the balance of Prediction and Evaluation functions in primates and rodents, respectively. Not only did these divergences result in obvious differences in cytoarchitecture and connectivity, but they also likely shaped specializations in OFC and ACC function. While there may be general functions that can be studied in rats that have reliably translated to macaques (for which there is strong evidence of convergence), careful attention should be paid to functions that may not translate well, or for which there is poor evidence of convergence. Future investigation should take into account species’ foraging niches when designing or interpreting experiments and, more importantly, translating their findings to humans.
